# Prolonged Sitting Induces Elevated Blood Pressure in Healthy Young Men: A Randomized Crossover Trial

**DOI:** 10.7759/cureus.55224

**Published:** 2024-02-29

**Authors:** Hajime Tamiya, Megumi Hoshiai, Takuya Abe, Hiroaki Watanabe, Yutaka Fujii, Atsuhiro Tsubaki

**Affiliations:** 1 Institute for Human Movement and Medical Sciences, Niigata University of Health and Welfare, Niigata, JPN; 2 Department of Cardiovascular Medicine and Nephrology, Dokkyo Medical University Nikko Medical Center, Nikko, JPN; 3 Department of Clinical Engineering and Medical Technology, Niigata University of Health and Welfare, Niigata, JPN

**Keywords:** peripheral vascular resistance, lower leg fluid retention, sympathetic nerve activity, mean arterial pressure, diastolic blood pressure, prolonged sitting

## Abstract

Introduction

Prolonged sitting-induced blood pooling in the lower legs can increase blood pressure through increased sympathetic nerve activity and peripheral vascular resistance, an aspect that has been understudied as a primary outcome. This study compared the effects of prolonged sitting with those of prolonged supination on blood pressure in healthy young men.

Methods

This randomized crossover study included 16 healthy young men (mean age: 21.6 ± 0.7 years) who were randomly assigned to a three-hour supine (CON) or three-hour sitting (SIT) condition, followed by a washout period of at least one week. Systolic blood pressure (SBP), diastolic blood pressure (DBP), mean arterial pressure (MAP), heart rate (HR), low-frequency/high-frequency (LF/HF) ratio derived from heart rate variability, and leg circumference were measured at 60, 120, and 180 minutes from baseline. These indices were compared by two-way (time × conditions) analysis of variance (ANOVA).

Results

In the SIT condition, DBP, MAP, HR, LF/HF ratio, and leg circumference increased significantly over time (P < 0.05) and were significantly higher than those in the CON condition (P < 0.05). However, SBP showed no significant change over time and between conditions.

Conclusions

The findings indicate the involvement of sympathetic nerve activity and increased peripheral vascular resistance induced by fluid retention in the lower legs with increased DBP and MAP in healthy young men.

## Introduction

Rapid technological advances, such as increased teleworking and expanded entertainment options at home, have increased our sedentary time per day [1]. Prolonged sitting is typically defined as sitting continuously uninterrupted for an hour or more at a time [2]. Previous studies have reported that prolonged sitting increases the risk of cardiovascular disease (CVD) and all-cause mortality [3,4], independent of body mass index (BMI) and physical activity [3]. In short, the sitting posture could have established a physiological environment distinct from that established in the body inactivity model (defined as less than 150 minutes of moderate to vigorous physical activity per week) and other experimental inactivity models [5], and numerous experimental studies conducted worldwide have sought to identify the underlying mechanism. A meta-analysis of such experimental studies revealed that prolonged sitting decreases the endothelial function of lower extremity vessels by 2.12% [6], which was associated with decreased arterial shear stress [7]. Such findings have been suggested as the mechanisms driving the increased risk of CVD and all-cause mortality.

Most experimental studies on prolonged sitting have focused on vascular function in the lower extremities. In contrast, only a few studies have examined the acute effects of prolonged sitting on blood pressure (the supposed primary outcome); this gap has led to questions about whether prolonged sitting influences blood pressure directly [1]. Notably, a recently published meta-analysis investigating the effect of sitting interruption on blood pressure showed that sitting interruption had no effect on systolic blood pressure (SBP); however, the effect on diastolic blood pressure (DBP) was poorly reported and insufficient for a meta-analysis [8]. Additionally, two previous reviews have noted that the effect of blood pressure was excluded from meta-analyses because a few studies have investigated the effects of sedentary interruptions and evaluated their effects on blood pressure [8-10]. Therefore, there is still a lack of evidence regarding the effect of prolonged sitting on blood pressure (especially DBP). This lack of evidence on the cardiovascular effects of prolonged sitting is a potential reason why specific guidelines for prolonged sitting remain undeveloped [11]. Hypertension, particularly, is a significant risk factor for CVD and a major cause of morbidity and mortality globally [12,13]. Therefore, understanding the effects of prolonged sitting on blood pressure is essential for elucidating how prolonged sitting affects the risks of CVD and all-cause mortality and to develop future guidelines for prolonged sitting.

Previous studies have reported that prolonged sitting may lead to fluid retention in the lower legs [2,14,15]. We hypothesized that excessive fluid retention in the lower extremities associated with prolonged sitting would cause a sustained increase in blood pressure via a reflex increase in centrifugal sympathetic nerve activity and peripheral vascular resistance. This study aimed to clarify the effects of prolonged sitting on blood pressure and elucidate the potential mechanisms.

## Materials and methods

Study design

This was a randomized, controlled, crossover trial, and the conditions were administered in a randomized order to minimize subjective bias. Participants were allocated randomly to the supine (CON, n = 16) or sitting (SIT, n = 16) position, with each maintained for three hours. The conditions were switched after a washout period of at least one week. Meta-analysis has shown that prolonged sitting for up to three hours progressively decreases peripheral hemodynamics [16]. Therefore, we set the experimental condition to three hours. The washout period was determined according to previous studies [7].

Study participants

The inclusion criteria for the present study were as follows: male participants aged between 18 and 30 years with a BMI of >18.5 and <25.0 kg/m^2^. The exclusion criteria were current smoking, history of CVD or metabolic disease, oral medications or dietary supplements, and moderate physical activity > 150 min/week and high intensity physical activity > 75 min/week. [17]. The criterion associated with physical activity was established to eliminate the possibility of habitual activity levels affecting blood pressure. Participation was voluntary. The absence of comorbidities in the participants was confirmed by self-report. A short form of the International Physical Activity Questionnaire (IPAQ) was used in this study to evaluate physical activity [18]. A total of 16 healthy young men participated in the study (mean age, 21.6 ± 0.7 years; height, 1.69 ± 0.04 m; weight, 60.1 ± 8.7 kg).

This study was conducted following the principles of the Declaration of Helsinki and was approved by the Ethics Committee of Niigata University of Health and Welfare (approval number: 18756-211126). It is also registered with the University Hospital Medical Information Network Center (UMIN trial ID: UMIN000052730). All participants provided written informed consent before enrolment into the study. The baseline characteristics of the study participants are shown in Table 1.

**Table 1:** Participant demographics Data are presented as the mean ± standard deviation BMI, body mass index; SBP, systolic blood pressure; DBP, diastolic blood pressure; MAP, mean arterial pressure; HR, heart rate; LF/HF, low frequency/high frequency; MVPA, moderate to vigorous physical activity; VPA, vigorous physical activity; bpm, beats per minute

Variable	Value (range)
N	16
Age (years)	21.6 ± 0.7 (21-23)
Height (m)	1.69 ± 0.04 (1.62-1.77)
Weight (kg)	60.5 ± 6.7 (52.0-66.8)
BMI (kg/m^2^)	21.2 ± 2.4 (18.7-24.6)
SBP (mmHg)	118.5 ± 10.3 (98-133)
DBP (mmHg)	70.8 ± 8.8 (54-89)
MAP (mmHg)	86.7 ± 7.9 (71.0-102.3)
HR (bpm)	68.1 ± 14.9 (53-102)
LF/HF ratio	3.4 ± 2.5 (1.0-10.3)
MVPA (minute/week)	13.1 ± 31.8 (0-120)
VPA (minute/week)	1.9 ± 7.3 (0-30)

Experimental procedures

**Figure 1:**
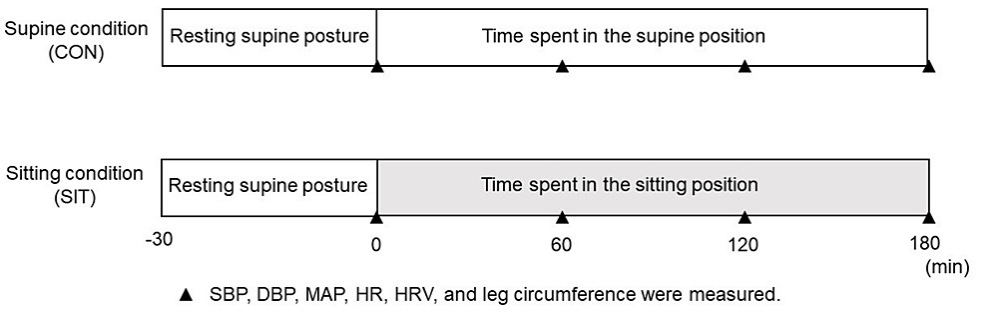
Two experimental conditions and measurement indices Two experimental conditions were used in this study: the supine condition (CON) involving 30 minutes of resting in a supine posture, followed by three hours in a supine position, and the sitting condition (SIT) involving 30 minutes of resting in a supine posture, followed by three hours in a sitting position. All measures were taken at baseline, followed by similar measurements at 60, 120, and 180 minutes SBP, systolic blood pressure; DBP, diastolic blood pressure; MAP, mean arterial pressure; HR, heart rate; HRV, heart rate variability

Figure 1 shows a schematic of the experimental design and the measurement timings for each indicator. The participants were instructed to eat a light meal at least two hours before arriving at the laboratory and refrain from caffeine, alcohol, and strenuous exercise 24 hours before arrival at the laboratory. All experiments were conducted at room temperature (24°C-26°C). The participants arrived between 8:00 a.m. and 9:00 a.m. and rested for 30 minutes in the supine resting position. Subsequently, the SBP and DBP in the brachial artery were measured thrice at each measurement timing (60, 120, and 180 minutes from baseline) using an automatic blood pressure monitor (HEM-8712, OMRON Healthcare, Kyoto, Japan), and the average value was adopted. The mean arterial pressure (MAP) was calculated from blood pressure recordings per convention: MAP = (SBP-DBP) / 3 + DBP.

Heart rate (HR) and heart rate variability (HRV) were measured using a three-lead electrocardiogram (LRR-03, GMS Co., Ltd., Tokyo, Japan) and the MemCalc/Bonaly Light software (GMS Co., Ltd. Tokyo, Japan) at a sampling frequency of 0.5 Hz, and the average value over two minutes was used. HRV parameters were calculated using low frequency (LF) and high frequency (HF), and LF/HF was calculated. LF reflects sympathetic and parasympathetic activities, representing baroreflex-mediated regulation, and was measured at 0.04-0.15 Hz [19]. HF is associated with parasympathetic or vagal activity and was measured at 0.15-0.4 Hz [20], and LF/HF is considered an indicator of autonomic balance [21].

The maximum lower leg circumference was measured as an index of venous pooling during prolonged sitting [21]. The lower leg circumference was measured at the maximum bulge of the gastrocnemius muscle. To efficiently minimize measurement bias, a nylon line (130 lb/0.98 mm, VARIVAS Co., Ltd., Saitama, Japan) without a scale was wrapped around the lower leg, a mark was made, and the distance between the marks was measured with a tape measure. Similarly, marks were made with flexible colored tape to enable repeated measurements at the same position [22]. Three measurements were taken each time, and the average value was used for analyses. During the experiment, the chair or bed was confirmed to not compress the lower leg.

The participants were prohibited from moving their lower legs or performing sympathetic activating movements (e.g., watching videos or playing games) throughout the experiment but were not restricted in their upper limb movements. The raters continuously monitored activity during the experiment.

Statistical analysis

Two-way (time × conditions) repeated measures analysis of variance (ANOVA) was performed, and Tukey's honestly test was conducted if significant differences were found. The GraphPad Prism 9.4.1 software (GraphPad Software, San Diego, CA) was used for statistical analysis, and the significance level was set at 5%. Data for the LF/HF ratio are presented as mean ± standard error, and other data are presented as mean ± standard deviation.

Sample sizes were calculated using the G*Power version 3.1.9.7 software (Heinrich-Heine-Universität Düsseldorf, Düsseldorf, Germany). A pilot study was conducted on six healthy young men, who were asked to recline in a supine position and then sit in an upright position for three hours each, and the change in MAP was measured to estimate the effect size. Based on the obtained effect size of 0.25, the sample size was calculated by setting α to 0.05 and 1-β to 0.8. As a result, the required sample size was 16 cases.

## Results

**Figure 2:**
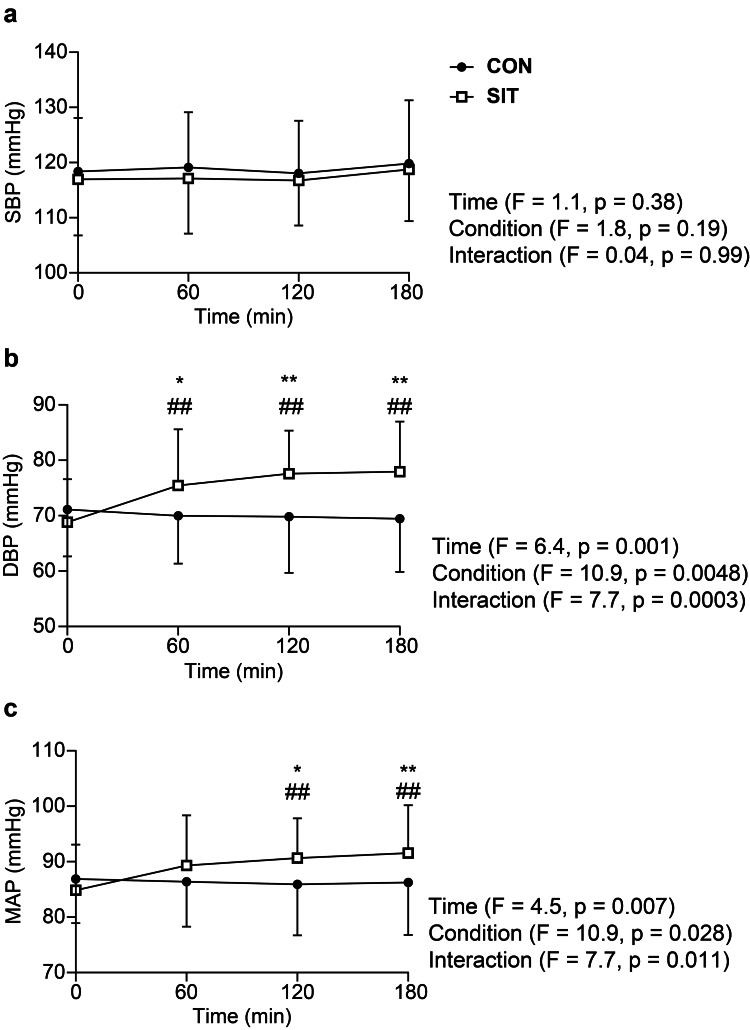
Changes in blood pressure over three hours in the sitting (SIT, n = 16) and supine (CON, n = 16) conditions Changes over time in SBP, DBP, and MAP. Error bars represent SD *Significant difference between conditions at P < 0.05 ##Significant difference from baseline at P < 0.01 **Significant difference between conditions at P < 0.01 SBP, systolic blood pressure; DBP, diastolic blood pressure; MAP, mean arterial pressure; SD, standard deviation

Changes in blood pressure over time are shown in Figure 2a-2c. Two-way ANOVA revealed insignificant interaction for SBP, with no significant increase over time in the two conditions (Figure 2a; P > 0.05). In contrast, a significant interaction was noted for DBP (Figure 2b; P < 0.01). In SIT, DBP was significantly increased from 60 to 180 minutes compared with that at baseline (P < 0.01) and was significantly higher than that in CON from 60 to 180 minutes (P < 0.05). Similarly, a significant interaction was found for MAP (Figure 2c; P < 0.05). In SIT, DBP was significantly increased from 120 to 180 minutes compared with that at baseline (P < 0.01) and was significantly higher than that in CON from 120 to 180 minutes (P < 0.05). Conversely, DBP and MAP in CON showed insignificant changes over time.

**Figure 3:**
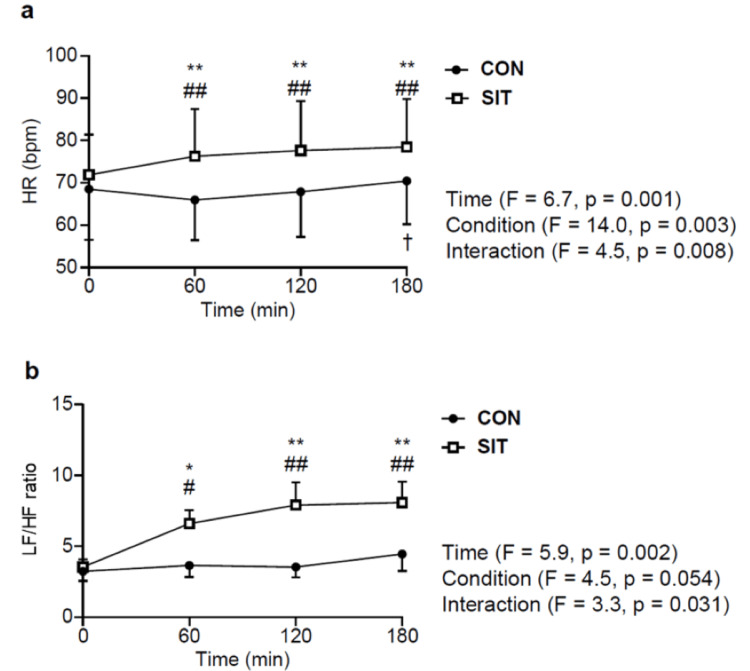
Changes in HR and low-frequency/high-frequency (LF/HF) ratio over three hours in the sitting (SIT, n = 16) and supine (CON, n = 16) conditions (a) Change in HR, with the error bars represented by SD. (b) LF/HF variation, with the error bars represented by SE #Significant difference from baseline at P < 0.05 *Significant difference between conditions at P < 0.05 ##Significant difference from baseline at P < 0.01 **Significant difference between conditions at P < 0.01 †Significant difference from 60 minutes after at P < 0.05 HR, heart rate; SD, standard deviation; SE, standard error; bpm, beats per minute

Changes in HR and LF/HF ratio over time are depicted in Figures 3a, 3b. Two-way ANOVA revealed a significant interaction for HR (Figure 3a; P < 0.01), demonstrating a significant increase from 60 to 180 minutes in SIT compared with that at baseline (P < 0.01) and a markedly higher value than that in CON from 60 to 180 minutes (P < 0.01). In CON, a significant increase was noted in HR from 60 to 180 minutes (P < 0.05). Similarly, a significant interaction was observed for the change in LF/HF over time (Figure 3b; P < 0.05). In SIT, the LF/HF ratio also exhibited a significant increase from 60 to 180 minutes compared with that at baseline (Figure 3b; P < 0.05) and was significantly higher than that in CON from 60 to 180 minutes (P < 0.05). Conversely, no significant changes over time were noted in CON.

**Figure 4:**
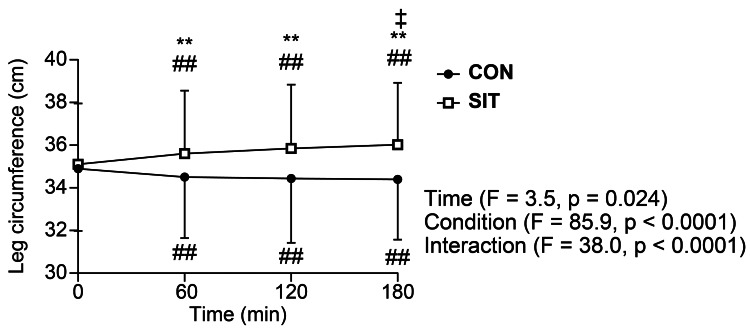
Changes in leg circumference over three hours in the sitting (SIT, n = 16) and supine (CON, n = 16) conditions Error bars represent SD ##Significant difference from baseline at P < 0.01 **Significant difference between conditions at P < 0.01 ‡Significant difference from 60 minutes after at P < 0.01 SD: standard deviation

Changes in leg circumference over time are shown in Figure 4. Two-way ANOVA revealed a significant interaction for leg circumference (Figure 4; P < 0.01), with a significant increase in SIT at 60-180 minutes compared with that at baseline (P < 0.01) and from 60 to 180 minutes (P < 0.05). In addition, leg circumference at 60-180 minutes was significantly higher in SIT than in CON (P < 0.01). However, a significant decrease was noted at 60-180 minutes in CON compared with that at baseline (P < 0.01).

## Discussion

In this study, we explored the effects of prolonged sitting on blood pressure and revealed a possible mechanism. The results showed that in healthy young men, prolonged sitting causes an increase in lower leg circumference and sympathetic nerve activity and induces increases in DBP and MAP, which are consistent with our hypothesis. To the best of our knowledge, this is the first study to investigate possible mechanisms underlying the effects of prolonged sitting as the primary outcome based on changes in blood pressure over time. The observed increase in DBP and MAP during prolonged sitting may be attributed to the increase in sympathetic nerve activity and peripheral vascular resistance that occurs with a decrease in venous return.

Prolonged sitting causes fluid retention in the lower legs [2,15]. In the present study, prolonged sitting increased lower leg circumference significantly over time (Figure 4), which is consistent with previous study findings. Fluid retention in the lower legs decreases venous return, further decreasing cardiac output [23]. Carotid baroreceptors rapidly detect this reduction in cardiac output, triggering the release of noradrenaline from the nerve endings of posterior sympathetic ganglion fibers, which in turn augments peripheral vascular resistance.

The increase in LF/HF ratio in the present study suggests sympathetic activation associated with these responses. A previous study also showed a significant increase in the LF/HF ratio after 1.5 hours of prolonged sitting, suggesting that the baroreflex was activated in response to hypotensive stimuli secondary to the progression of lower extremity blood retention [21]. The fact that the LF/HF ratio did not increase or decrease significantly over time in the CON treatment in the present study (i.e., the three-hour supine rest condition) strongly suggests that the response was specific to the sitting posture. Conversely, Headid et al. [2] reported that the LF/HF ratio did not increase after 2.5 hours of prolonged sitting, which may be due to the participants' high physical activity (approximately 12,000 steps/day) and high exercise tolerance (the average maximal oxygen consumption {V̇O_2_max} in these participants was 45.9 ± 9.3 mL/kg/minute). This suggests that the effects on autonomic nervous system activity during prolonged sitting may differ depending on the participants' physical activity level and exercise tolerance. Therefore, it is necessary to expand the range of participants and clarify the differences between active and inactive participants in the future.

Previous research has indicated that DBP depends on total peripheral vascular resistance and arterial compliance [24]. Therefore, any increase in total peripheral vascular resistance contributes to elevated DBP. In contrast, reduced arterial compliance leads to a decrease in DBP [25], indicating that changes in DBP in this study were influenced significantly by total peripheral vascular resistance. The persistence of leg edema from prolonged sitting unloads the carotid baroreceptors, further contributing to nocturnal blood pressure elevation by reducing baroreceptor afferent activity and consequently inducing a reflex increase in centrifugal sympathetic activity [1]. Similarly, recent studies have shown the involvement of such baroreceptor reflexes and the sympathetic nervous system in short-term and long-term blood pressure regulations [26-28]. Therefore, chronic conditions of leg edema caused by prolonged sitting may increase the risk of hypertension, contributing to an increased risk of the development of CVD and all-cause mortality.

However, SBP did not change significantly between CON and SIT, possibly because the participants maintained sufficient cardiac output by increasing HR, and the elasticity of their blood vessels was sufficiently preserved. Considering that arterial stiffness is strongly associated with SBP [29], prolonged sitting may increase SBP more easily in individuals with arterial stiffness compared with those without arterial stiffness.

The present study had certain limitations. First, the study was limited to healthy young men, making it challenging to extrapolate the findings to other demographic populations. Second, stroke volume and peripheral vascular resistance were not measured directly. Third, in this study, only the amount of physical activity was used to assess individual behavioral habits. In future studies, regression analysis with various confounding factors may be able to determine whether prolonged sitting is an independent factor in the outcome.

Despite such limitations, the present study makes two significant contributions by demonstrating the effect of prolonged sitting on blood pressure. First, the increase in MAP was attributed to increased DBP, which reflects peripheral vascular resistance, suggesting that the increased blood pressure can be suppressed by decreasing peripheral vascular resistance during prolonged sitting. Second, DBP increased significantly after 60 minutes. Previous guidelines on sitting time do not indicate when to discontinue prolonged sitting [30]. However, the results of the present study suggest that interrupting prolonged sitting once every 30 minutes may be an appropriate approach to reducing blood pressure elevation. For example, interventions promoting muscle pumping in the lower legs, such as short walks and leg raises, could be beneficial. In the future, the scope of this study should be expanded, and effective preventive methods should be verified for blood pressure control. The study findings provide fundamental insights into public health concerns regarding prolonged sitting and its association with an increased risk of CVD, thus contributing to the development of guidelines for managing prolonged sitting.

## Conclusions

This study demonstrated that prolonged sitting significantly increased DBP and MAP over time in healthy young men owing to increased sympathetic nerve activity and peripheral vascular resistance induced by fluid retention in the lower legs.

The results suggest that improving fluid retention in the lower extremities during prolonged sitting can prevent blood pressure elevation. Furthermore, a significant increase in DBP was observed after one hour of sitting, indicating that continuous sitting should be interrupted at least once within one hour. However, since the participants in this study were healthy young men, future studies should examine the effects of differences between the sexes and between different diseases.
